# Effect of perioperative ultrasound guided fascia iliaca compartment block in elderly adults with hip fractures undergoing arthroplasty in spinal anesthesia—a randomized controlled trial

**DOI:** 10.1186/s12877-023-03786-5

**Published:** 2023-02-02

**Authors:** Liang Chen, Shuangmei Liu, Yanyan Cao, Lei Yan, Yang Shen

**Affiliations:** 1 Department of Anesthesiology, Shengjing Hospital of China Medical University, No.36 Sanhao Street, Heping District, Shenyang, 110004 Liaoning Province China; 2 Department of Emergency Medicine, Shengjing Hospital of China Medical University, No.36 Sanhao Street, Heping District, Shenyang, 110004 Liaoning Province China

**Keywords:** Hip fracture, Hip arthroplasty, Fascia iliaca compartment block, Spinal anesthesia, Elderly adults

## Abstract

**Background:**

For elderly adults undergoing hip arthroplasty, fascia iliaca compartment block (FICB) is often used before spinal anesthesia to reduce the pain of posture placement. However, the impact of FICB within 48 h needs further study.

**Methods:**

89 elderly adults scheduled to undergo arthroplasty for hip fracture were enrolled and randomized into the FICB group (*n* = 45) and the control group (*n* = 44). The fascia iliaca on the operated side was located using ultrasound, and a puncture needle was placed under the fascia iliaca. The FICB group was injected with 40 ml of 0.5% ropivacaine, and the control group was injected with 40 ml of normal saline. Spinal anesthesia was performed after 20 min. Our primary outcome measures were: duration of analgesia, muscle strength, and Quality of Recovery (QoR).

**Results:**

The duration of analgesia in the FICB group was 403.5 ± 39.6 min, which was longer than that (357.5 ± 35.9 min) of the control group (*P* = 0.012). There were 19 (42.2%) patients with muscle strength of grade 4 in the FICB group and 36 (81.8%) patients with muscle strength of grade 4 in the control group. FICB group was lower (*P* < 0.001). QoR-15 at 24 h after surgery was 114.1 ± 8.3 in the FICB group and 104.6 ± 8.4 in the control group (*P* < 0.001). QoR-15 at 48 h after surgery was 122.7 ± 8.4 in the FICB group and 120.5 ± 9.5 in the control group (*P* = 0.232).

**Conclusions:**

For elderly adults with hip fractures, FICB provided longer analgesia and improved 24-h QoR, but reduced postoperative muscle strength.

**Trail registration:**

Chinese Clinical Registry Center, ChiCTR2200056937, 23/02/2022.

## Introduction

As the global population is aging, hip fracture is becoming an increasingly serious public health problem [1]. It is a leading cause of serious morbidity in individuals aged 65 years and older [2]. By 2050, the global number of hip fractures is expected to increase to 4.5 million and incur huge medical and social costs [3].

Displaced femoral neck fractures are usually an indication of early surgical intervention [1]. Current evidence supports the superiority of arthroplasty over internal fixation, particularly in the population 65 years of age and older[4].Currently, more than one million hip arthroplasty surgeries are performed worldwide each year, and this number is expected to double by 2030 [5].

Spinal anesthesia is a common anesthesia administration for hip arthroplasty due to its advantages of reduced operative time, less bleeding, and fewer complications [6, 7]. It is difficult for patients with hip fractures to remain in a lateral or sitting position required for spinal anesthesia. The use of fascia iliaca compartment block (FICB) before spinal anesthesia has been confirmed to be an effective analgesic method for not only reducing the positioning pain but also shortening the puncture time of spinal anesthesia [8, 9]. However, no detailed research has been reported on whether the use of FICB still has a lasting effect on patients after spinal anesthesia within 48 h. In particular, whether it has influence on the postoperative recovery of patients. This study aimed to find the subsequent effects of FICB by a randomized controlled trial.

## Methods

### Study design and ethics

This study was designed as a randomized, controlled trial. Ethical approval for this study (approval number 2021PS511K) was provided by the Institutional Review Board of Shengjing Hospital, Shenyang, China (Chairperson Prof Y. Zhao) on 12 May 2021. The trial was registered with the Chinese Clinical Registry Center (registration No. ChiCTR2200056937, dated 23/02/2022) before patient enrollment. This study was conducted in the Shengjing Hospital of China Medical University (Shenyang, China). Written informed consent was obtained from all subjects participating in the trial. The trial protocol is following the Declaration of Helsinki. This manuscript adheres to the applicable CONSORT guidelines.

### Inclusion and exclusion criteria

Inclusion criteria: patients over 65 years of age about to undergo primary hip arthroplasty under spinal anesthesia.

Exclusion criteria: patients unable to walk independently before the injury; patients with fractures in other parts; patients with neurological diseases (for example, Alzheimer’s disease or dementia); patients with contraindications to regional anesthesia (for example, local infection, coagulation abnormalities, or patient refusal); patients allergic to study medications.

### Randomization and blinding

A computer-generated random allocation sequence was created by an independent investigator using SPSS Statistics (version 24.0, IBM Corp., Armonk, NY, USA) with a 1: 1 allocation and random block size. Recruitment was performed by the same investigator. After providing written informed consent, the participants were randomized to the FICB group and control group, using sealed opaque envelopes to reveal the treatment arm on the morning of surgery.

None of the patients, anesthesiologists responsible for injection under the subfascial iliaca, or data collectors were aware of the grouping. Only one staff (Dr. Shen) among the researchers was aware of the grouping. Her task was to recruit and randomize patients and to assign study drugs to anesthesiologists based on the grouping.

### Anesthetic techniques and interventions

After the patient was admitted to the operating room, the patient received an injection under the fascial iliaca in the supine position, which was performed by a designated physician. The patient was placed in the supine position under bedside ultrasound guidance using a high-frequency linear-array probe. After disinfection of the skin in the inguinal region and upper thigh, the ultrasound probe was placed horizontally just under the inguinal ligament lateral to the femoral artery. The fascia lata and fascia iliaca appeared as two hyperechoic lines. A 22-G puncture needle was inserted into the lateral thigh and 1 cm beyond the edge of the probe. The needle was inserted from the lateral side to the medial side, using an in-plane technique. The tip of the needle was passed through the fascia lata and then through the fascia iliaca. After puncturing the fascia iliaca and application of negative pressure suction, a pre-assigned solution was injected, which was 40 mL 0.5% ropivacaine for the FICB group and 40 ml saline for the control group. An expanding anechoic shadow between the fascia iliaca and the iliopsoas muscle served as a visual confirmation of the correct injection of the drug solution.

Spinal anesthesia was performed 20 min after the completion of the drug injection. The patient was placed in the lateral position with the fracture side up. The intervertebral spaces between lumbar vertebrae L2-L3 and between lumbar vertebrae L3-L4 were located using ultrasound, and the anesthesiologists were free to select the appropriate intervertebral space for puncture. After confirming the free flow of cerebrospinal fluid, the intrathecal injection was performed using a 25-G spinal anesthesia needle with 1.5 to 2 mL of 0.5% isobaric bupivacaine. After administration of spinal anesthesia, the patient was changed to a supine position. Every one minute, the sensation was tested using a blunt-tipped needle, and anesthesia was considered adequate when the level of anesthesia reached T10. All subsequent surgical procedures were performed by three orthopedic surgeons with extensive experience in hip arthroplasty. As indicated by the hospital electronic medical record, all three participating orthopedic surgeons could routinely perform primary hip arthroplasty within 60–90 min, thereby ensuring the appropriateness of spinal anesthesia for the surgery.

### Intraoperative management

If the level of anesthesia was below T10, the patient was placed in a 20–30° head-down position. If it was still below T10 after 15 min, general anesthesia was performed, and the patient was excluded from the trial. If the anesthesia level was higher than T6, the patient was placed in a 20–30° head-up position while under close observation for the slowing of breathing or hypotension. If there was a drop in blood pressure (mean arterial pressure (MAP) below 60 mmHg or 70% of the basal value), the patient was treated with rapid fluid resuscitation and was administered with 10 mg ephedrine or 50 μg phenylephrine, depending on the heart rate. If the patient developed nausea and vomiting, 0.3 mg ramosetron was administered intravenously.

### Outcome measurements

#### Primary outcomes

##### P1. Duration of analgesia

It was defined as the duration from intrathecal injection of bupivacaine to the first time the patient complained of pain in the limb on the operated side.

##### P2. Muscle strength

The strength of the non-operative side quadriceps muscle was measured every 15 min after surgery, and when the muscle strength reached grade 4, the quadriceps muscle strength on the operated side was measured and recorded. Muscle strength was determined by testing the movement of the knee or hip joint. Muscle strength was graded as 0, no contraction; 1, muscle flicker; 2, active movement but not against gravity; 3, active movement against gravity; 4, the movement against some resistance; and 5, full strength against resistance.

##### P3. Quality of Recovery (QoR)

The QoR-15 was used to evaluate the QoR after surgery [10, 11]. QoR-15 consists of 15 items, with a total score of 0–150. The higher the score, the better the recovery. The questionnaire was conducted 24 h and 48 h after surgery.

#### Secondary outcomes

##### S1. Lowest MAP in different periods

The lowest blood pressure MAP1 was recorded before the injection under the subfascial iliaca, the lowest blood pressure MAP2 was recorded after the injection under the subfascial iliaca but before the spinal anesthesia, and the lowest intraoperative blood pressure MAP3 was recorded after the intrathecal bupivacaine injection until the end of the surgery.

##### S2. Time to perform spinal anesthesia and visual analogue scale (VAS) score during operation

Time to perform spinal anesthesia is defined as the time from the patient’s change of body position to the recovery to the supine position. When spinal anesthesia was completed, patients were asked to perform a VAS score to assess the most severe pain in the process.

##### S3. Onset time of anesthesia

It was defined as the time from the end of intrathecal bupivacaine injection to when the level of anesthesia reached T10, as confirmed by needle pricking in the midclavicular line on the operated side.

##### S4. Duration of anesthesia

The level of anesthesia was measured bilaterally in the midclavicular lines every 15 min after surgery, and the anesthetic effect was considered to have disappeared when the level of anesthesia on the operated side was below T10. The time from the intrathecal bupivacaine injection to the end of anesthesia was recorded.

##### S5. Complications

Complications caused by operation of FICB, such as bleeding, hematoma, or infection at the puncture site.

### Statistical analysis

#### Sample size calculations

According to the data obtained from the pre-test, we assumed that the average postoperative analgesia time in the FICB group was 400 min and 360 min in the control group, with a standard deviation of 60. Based on the abovementioned hypothesis, to achieve 90% statistical power (β = 0.1) with a two-sided confidence interval of 95% (α = 0.05), we used PASS11 to calculate that with a total sample size of 80 patients, each group should contain 40 patients. Considering the likelihood of screening failures and dropouts, we requested permission from the Institutional Review Board to enroll up to 25% more patients.

#### Statistical methods

The normality of the continuous data was tested using the Shapiro–Wilk test. Normally distributed data were expressed as a Mean ± SD, while non-normally distributed data were expressed as a Median (interquartile ranges [IQR] xx to yy). Count data were expressed as numbers or percentages. The between-group comparison was performed using an independent t-test for normally distributed measurement data, and the Mann–Whitney U test for non-normally distributed measurement data. For count data, the between-group comparison was performed using chi-square analysis. *P* < 0.05 was considered statistically significant. SPSS 24 software (IBM Corp., Armonk, NY, USA) was used for statistical analysis.

## Results

**Fig. 1:**
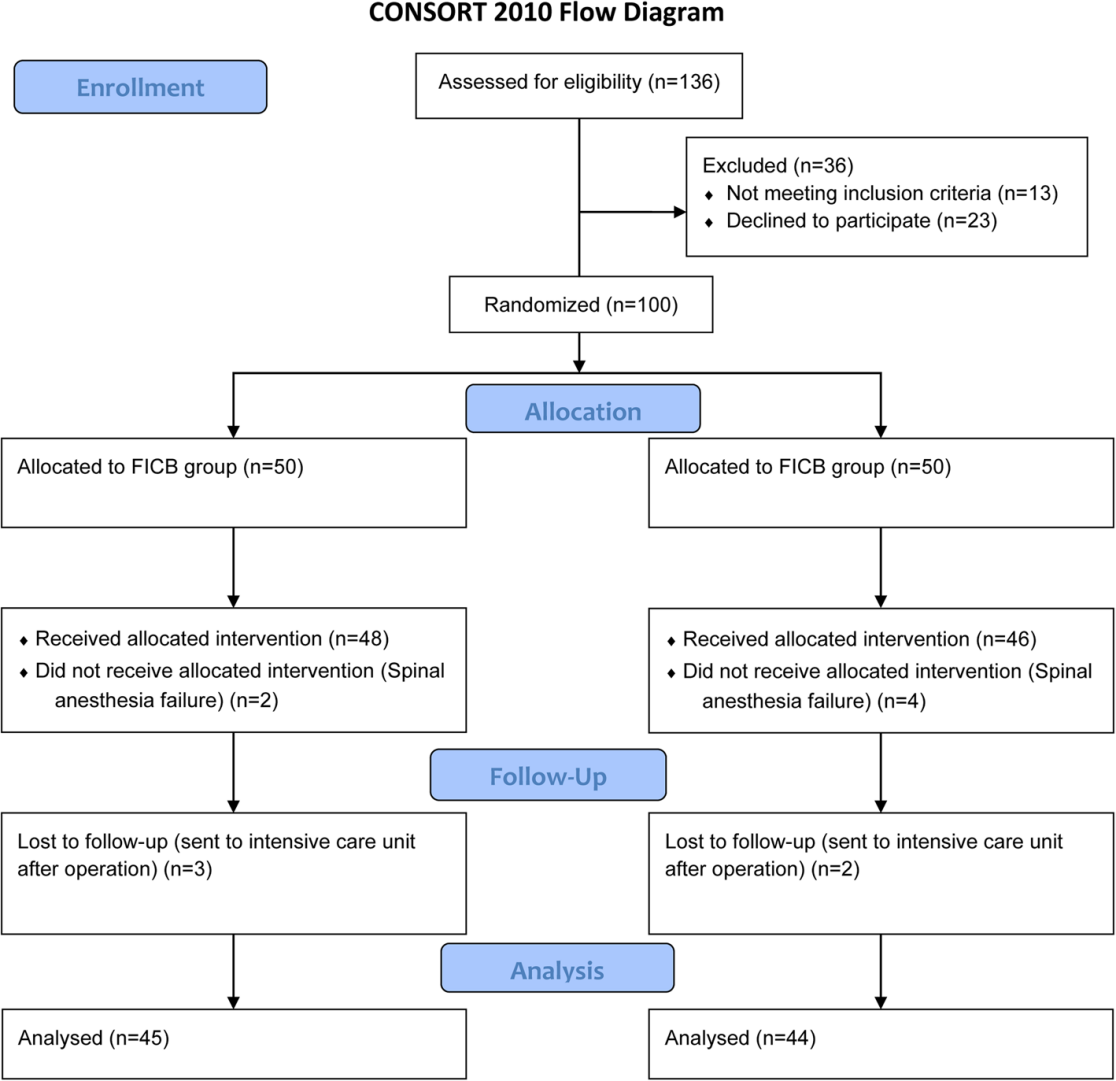
Consolidated Standards of Reporting Trials (CONSORT) flow diagram

A total of 100 patients were enrolled and randomized into a FICB group (*n* = 50) and a control group (*n* = 50) from March to August 2022. Of them, six were excluded due to failure of spinal anesthesia, and five were excluded due to loss of follow-up because of postoperative admission to the intensive care unit. As a result, 45 in the FICB group and 44 in the control group were finally involved in all aspects of the trial, and their data were analyzed (Fig. 1).

Through Shapiro Wilk test, the following data conform to normal distribution: body mass index、duration of analgesia、the QoR-15、duration of anesthesia、MAP1-3.

The following data were collected for all the 89 patients: age, gender, body mass index, ASA physical status, type of surgery, intervertebral space, duration of surgery, amount of intraoperative bleeding, and hospitalization days. There were no significant differences in the mentioned variables between the two groups of patients (Table 1).

**Table 1:** Patient Characteristics and Intraoperative Details

	FICB group *n* = 45	control group *n* = 44	test value	*P*-value
Age (yr), Median [IQR]	74 [70 to 78]	72 [67 to 77]	986.5	0.977
Male sex, N (%)	16 (35.6%)	18 (40.9%)	0.270	0.603
BMI (kg/m^2^), Mean ± SD	22.64 ± 2.60	22.12 ± 2.09	1.040	0.301
ASA physical status, N (%)			0.026	0.872
I	0 (0%)	0 (0%)		
II	31 (68.9%)	31 (70.5%)		
III	14 (31.1%)	13 (29.5%)		
IV	0 (0%)	0 (0%)		
Type of surgery, N (%)			1.001	0.317
Total hip arthroplasty	34 (75.6%)	29 (65.9%)		
Hemiarthroplasty	11 (24.4%)	15 (34.1%)		
Intervertebral space, N (%)			0.546	0.460
L23	25 (55.6%)	21 (47.7%)		
L34	20 (44.4%)	23 (52.3%)		
Surgery time (min), Median [IQR]	70 [63 to 77]	72.5 [63 to 82]	982.5	0.950
Bleeding volume (ml), Mean ± SD	300 ± 95	275 ± 155	909.0	0.504
Hospitalization days, Median [IQR]	8 [7 to 8]	7 [6.25 to 8]	772.5	0.062

*BMI* Body mass index, *ASA* American Society of Anesthesiologists, *SD* Standard deviation, *IQR* Interquartile range

The FICB group had a longer duration of analgesia (403.5 ± 39.6 min), which was statistically different from that of the control group (357.5 ± 35.9 min), (t = 5.742, *P* < 0.001).

In the FICB group, 19 patients (42.2%) had grade 4 postoperative muscle strength, with 26 patients (57.8%) showing postoperative muscle strength of grade 3. In the control group, 36 patients (81.8%) with postoperative muscle strength of grade 4, and 8 patients (18.2%) with postoperative muscle strength of grade 3. The difference between the two groups was statistically significant (χ2 = 14.775, *P* < 0.001).

24 h after surgery, QoR-15 in the FICB group was 114.1 ± 8.3 and 104.6 ± 8.4 in the control group. There was a difference between the two groups (t = 5.379, *P* < 0.001). 48 h after surgery, QoR-15 in the FICB group was 122.7 ± 8.4 and 120.5 ± 9.5 in the control group. There was no statistically significant difference between the two groups (t = 1.203, *P* = 0.232).

As detailed in Table 2, the time to perform spinal anesthesia in the FICB group was shorter than that in the control group, and the VAS score was lower than that in the control group. However, there was no statistically significant difference in the onset time of anesthesia and duration of anesthesia between the two groups. There was no statistically significant difference in MAP1 and MAP3 between the two groups. The MAP2 in the FICB group was significantly lower than control group.

**Table 2:** Observation indexes of spinal anesthesia in two groups

	FICB group *n* = 45	control group *n* = 44	test value	*P*-value
Time to perform spinal anesthesia (min), Median [IQR]	14 [12 to 18]	22 [18 to 27]	219.5	< 0.001
VAS score during operation of spinal anesthesia, Median [IQR]	3 [2 to 4]	4.5 [3.25 to 5.75]	313.5	< 0.001
onset time of anesthesia Median [IQR]	6 [5 to 7]	6 [5 to 7]	846.0	0.228
duration of anesthesia (min), Mean ± SD	172.4 ± 21.7	166.0 ± 22.4	1.368	0.175

*SD* Standard deviation, *IQR* Interquartile range

## Discussion

The trial revealed that the FICB group had a longer duration of analgesia and lower grade of muscle strength compared with the control group. The QoR score of the FICB group at 24 h after surgery was higher than that of the control group, and there was no statistically significant difference between the two groups at 48 h after surgery. Meanwhile, the lowest MAP before spinal anesthesia was significantly lower in the FICB group than in the control group. The time to perform spinal anesthesia was shorter and the VAS score was lower in the FICB group. There is no significant between-group difference in the onset time and duration of spinal anesthesia.

It is beneficial to complete FICB before spinal anesthesia. Proper positioning of the lumbar spine is a prerequisite for successful neuraxial anesthesia, but it is difficult to achieve due to the pain at the fracture site [12]. FICB before spinal anesthesia can relieve the pain of positioning and improve the position of the patients with broad patient acceptance [13]. This is consistent with our study results.

Spinal anesthesia is usually judged by the sensation of the skin. The onset of anesthesia was defined as when the level of anesthesia on the operated side reached T10, as the T10 level is the required level of anesthesia for hip arthroplasty surgery [14]. Correspondingly, the end of anesthesia was defined as when the level of anesthesia on the operated side dropped below T10. The results of our study show that there was no difference in spinal anesthesia related indexes such as onset time and duration between the two groups. The results confirmed that the sensory block produced by FICB would not affect the judgment of spinal anesthesia.

The longer duration of analgesia in the FICB group should be directly attributed to the FICB rather than to a prolongation of the analgesic effect of spinal anesthesia. Previous trials have shown that the use of FICB can provide effective postoperative analgesia for patients undergoing hip arthroplasty and facilitate the maintenance of hemodynamic stability [15, 16]. Effective analgesia can improve the postoperative recovery of patients.

The QoR-15 provides a valid, extensive, and efficient evaluation of postoperative QoR [10, 11]. Previous studies have shown that the minimum difference in clinical importance of QoR-15 is 8 [17]. The difference in the mean QoR-15 at 24 h after surgery between the two groups was 10, indicating that FICB had an impact on the recovery of patients 24 h after surgery. However, there was no difference in QOR-15 48 h after surgery. The difference in QoR-15 between the two days suggested that the difference in the quality of recovery may come from the longer postoperative analgesia provided by FICB on the postoperative day.

We found in our research that FICB may also have adverse effects. The trial revealed that patients in FICB group had lower postoperative muscle strength in the limb on the side of surgery than in control group. Assuming the absence of the effect of FICB, when the muscle strength of the contralateral lower limb recovered to grade 4, the muscle strength of the operated side should have recovered to grade 4 as well. This suggested that the FICB decreased the quadriceps muscle strength even after the effect of spinal anesthesia on muscle strength had disappeared. This finding is similar to a previous one in which the FICB group injected with 40 mL of 0.2% ropivacaine showed lower postoperative quadriceps muscle strength and a higher risk of falls than the control group injected with normal saline [18]. Such an effect of FICB may not matter for patients who need to be bedridden. However, for patients who need early ground activities and start rehabilitation training as soon as possible, attention should be paid to preventing falls. Early ambulation on the day of operation or the next day is an important index of rehabilitation in patients with hip arthroplasty [19, 20]. Using short-acting or low-concentration local anesthetics to realize FICB can prevent the decline of muscle strength, but it will also weaken postoperative analgesia.

Previous trials have concluded that FICB does not affect the blood pressure in patients [12]. However, our study revealed a between-group difference in the lowest blood pressure after FICB but before spinal anesthesia, with the FICB group having a lower lowest blood pressure. One possible explanation for this difference is that during the puncture of spinal anesthesia, FICB group had analgesia, while the control group had no analgesia. The blood pressure of the control group was higher than that of FICB group because of the pain stimulus caused by puncture. This suggests that patients should be carefully monitored after FICB, especially those who are critically ill or at high risk of hypotension [21]. However, FICB did not exacerbate hypotension after spinal anesthesia, and there was no difference in intraoperative hypotension between the two groups, thereby suggesting the safety of FICB.

### Limitations

1. This study restricted the study population to elderly hip fracture patients undergoing hip arthroplasty under spinal anesthesia. For other relevant populations, such as elderly patients undergoing surgery under general anesthesia or undergoing internal fracture fixation, the results of this study may be applicable, but further studies are needed. 2. The evaluation of postoperative analgesia was based on the subjective feelings of patients. The data of post-operative opioids consumption were not collected. 3. This study was followed up to 48 h after the operation. Further research is needed on the impact of a longer time, such as the impact on the incidence of postoperative cognitive function and postoperative delirium.

## Conclusions

The use of perioperative FICB is safe in elderly adults with hip fractures undergoing arthroplasty in spinal anesthesia. FICB provides longer duration of analgesia and improves 24-h QoR. However, it is worth noting that FICB may decrease muscle strength after operation and lead to hypotension before spinal anesthesia.

## Data Availability

The research data has been uploaded to the database. The public access to the database is open. https://doi.org/10.6084/m9.figshare.20479068. The datasets used and/or analysed during the current study are available from the corresponding author on reasonable request.

## Abbreviations

FICB: Fascia iliaca compartment block
QoR: Quality of Recovery
MAP: Mean arterial pressure
VAS: Visual analogue scale
ASA: American Society of Anesthesiologists
BMI: Body mass index
SD: Standard deviation
IQR: Interquartile range
